# A comprehensive overview of psoriatic research over the past 20 years: machine learning-based bibliometric analysis

**DOI:** 10.3389/fimmu.2023.1272080

**Published:** 2023-10-26

**Authors:** Chenyang Yu, Yingzhao Huang, Wei Yan, Xian Jiang

**Affiliations:** ^1^ Department of Dermatology, West China Hospital, Sichuan University, Chengdu, China; ^2^ Laboratory of Dermatology, Clinical Institute of Inflammation and Immunology, Frontiers Science Center for Disease-related Molecular Network, West China Hospital, Sichuan University, Chengdu, China; ^3^ Department of Thoracic Surgery, Sichuan Cancer Hospital and Institute, Sichuan Cancer Center, School of Medicine, University of Electronic Science and Technology of China, Chengdu, Sichuan, China

**Keywords:** psoriasis, bibliometric, machine learning, natural language processing (NLP), latent Dirichlet allocation (LDA) algorithm

## Abstract

**Background:**

The surge in the number of publications on psoriasis has posed significant challenges for researchers in effectively managing the vast amount of information. However, due to the lack of tools to process metadata, no comprehensive bibliometric analysis has been conducted.

**Objectives:**

This study is to evaluate the trends and current hotspots of psoriatic research from a macroscopic perspective through a bibliometric analysis assisted by machine learning based semantic analysis.

**Methods:**

Publications indexed under the Medical Subject Headings (MeSH) term “Psoriasis” from 2003 to 2022 were extracted from PubMed. The generative statistical algorithm latent Dirichlet allocation (LDA) was applied to identify specific topics and trends based on abstracts. The unsupervised Louvain algorithm was used to establish a network identifying relationships between topics.

**Results:**

A total of 28,178 publications were identified. The publications were derived from 176 countries, with United States, China, and Italy being the top three countries. For the term “psoriasis”, 9,183 MeSH terms appeared 337,545 times. Among them, MeSH term “Severity of illness index”, “Treatment outcome”, “Dermatologic agents” occur most frequently. A total of 21,928 publications were included in LDA algorithm, which identified three main areas and 50 branched topics, with “Molecular pathogenesis”, “Clinical trials”, and “Skin inflammation” being the most increased topics. LDA networks identified “Skin inflammation” was tightly associated with “Molecular pathogenesis” and “Biological agents”. “Nail psoriasis” and “Epidemiological study” have presented as new research hotspots, and attention on topics of comorbidities, including “Cardiovascular comorbidities”, “Psoriatic arthritis”, “Obesity” and “Psychological disorders” have increased gradually.

**Conclusions:**

Research on psoriasis is flourishing, with molecular pathogenesis, skin inflammation, and clinical trials being the current hotspots. The strong association between skin inflammation and biologic agents indicated the effective translation between basic research and clinical application in psoriasis. Besides, nail psoriasis, epidemiological study and comorbidities of psoriasis also draw increased attention.

## Introduction

Psoriasis is a common, chronic, and inflammatory disease characterized by erythematous and scaly skin lesions, affecting over 60 million people in the world (1, 2). The prevalence of psoriasis is reported to be 0.1%-1.5%, and higher in western counties (1, 2). The World Health Organization has recognized psoriasis as a chronic, painful, disfiguring, and disabling disease in 2014, due to the substantial burden that it imposes on individuals and the whole society (3). Patients with psoriasis suffer from various associated systemic disorders, including psychological disorders, metabolic syndrome (MetS), and cardiovascular diseases (CVD). Psoriasis is genetically predetermined, with immunological factors and environmental triggers contributing to its development (4). The central pathological mechanism is the abnormal crosstalk between innate and adaptive immune systems (5). Conventional therapies include topical agents, phototherapy, and oral agents (methotrexate, ciclosporin, acitretin, fumarates). Over the past two decades, biological agents have dramatically changed the landscape of treating psoriasis. Tumor necrosis factor alpha (TNF-α) and components of the interleukin (IL)-23/IL-17 axis are major therapeutic targets (6).

Owing to substantial advancements in research methodologies, a notable surge in the volume of scholarly articles pertaining to psoriasis has occurred in recent years. In the year of 2003, only 689 publications regarding psoriasis were published, and the number increased to 2318 in 2022. It is difficult for researchers to deal with the enormous literature. New methods are needed to describe and summarize the current research hotspots and trends of metadata. Bibliometrics is the quantitative study of academic publications that applies statistics to discover publishing trends, identify correlations between published works, and identify current hotspots. However, due to the lack of tools to deal with metadata, previously published bibliometric analysis on psoriasis may focus on a specific subtype (nail psoriasis (7), psoriatic arthritis (8)), a specific region (9), comorbidities (10), or the 100 top-cited articles (11). No comprehensive bibliometric analysis on psoriasis was conducted based on our knowledge.

Natural Language Processing (NLP) stands as a pivotal computational approach widely adopted for dissecting and interpreting human language. Applying NLP to processing large-scale medical information has yielded substantial research achievements (12). Latent Dirichlet allocation (LDA), which is an unsupervised learning algorithm, can process and screen the large datasets of unstructured text through creating a term function vocabulary. The present study aims to provide a gross landscape and identify the recent trends of psoriatic research from a macroscopic perspective, by analyzing publications in psoriatic research from January 2002 to December 2022 via machine learning methods.

## Materials and methods

### Screening of publications and access

All PubMed publications from 1^st^ January, 2002 to 31^st^ December, 2022 were searched with the Medical Subject Headings (MeSH) (https://www.ncbi.nlm.nih.gov/mesh) term “Psoriasis”. We used the R (https://www.r-project.org/, version: 4.1.2) package easyPubMed (https://cran.rproject.org/web/packages/easyPubMed/index.html) to download the metadata. A complete record of the search results is downloaded in XML format, which contains publication data, title, abstract, MeSH terms of each article. Visualizations and data analysis are mostly based on Python (https://www.python.org/, version: 3.10.0).

### Latent Dirichlet allocation analysis

LDA is a probabilistic graphical model used for topic modeling of text collections. It is an unsupervised learning algorithm that can automatically discover topics from large-scale literature without the need for manual annotations. In LDA, each document is treated as a mixture of multiple topics, where each topic is assumed to be a probability distribution over multiple words. We used LDA model to find the most likely combination of topics that can explain the word distribution in each article’s abstract. The number of topics was set to 50, which was based on appropriate perplexity, redundancy, and legibility, as well as previous studies (13–16). We used the LDA algorithm to calculate the probability of each topic in each article, and based on this probability, we identified the primary topic of each article as the one with the highest probability score. For each category, 30 to 50 articles were randomly selected. Then, two authors (Chenyang Yu and Yingzhao Huang) manually named the topic of each category independently by reviewing the keywords generated by LDA algorithm, as well as titles and abstracts of the selected articles. Any dissensions between the two authors were resolved by the third author (Wei Yan).

To analyze the relationships between prominent topics of interest, we determined the top two topics for each article based on their probability of attribution, counted the frequency of each topic in each document, and then established a connection between the topics. The Louvain algorithm in Gephi software (https://gephi.org/, version 0.10) were used for clustering analysis.

## Results

### The number of publications in psoriasis research increases every year

**Figure 1:**
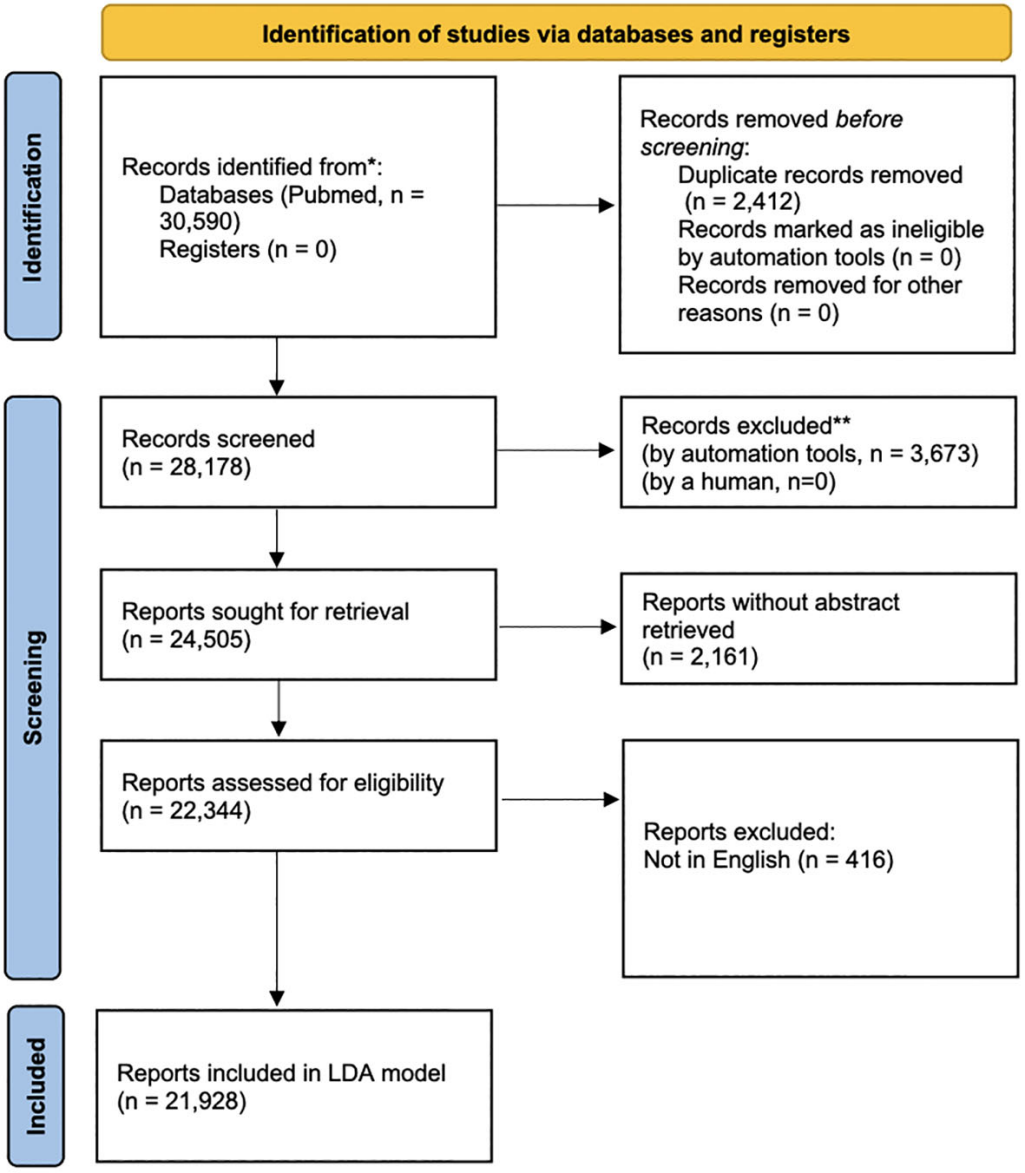
The flowchart of screening the publications on psoriasis in the past 20 years. PubMed publications on MeSH term “psoriasis” from 1^st^ January, 2003 to 31^st^ November, 2022 were screened and downloaded. 30,590 publications were initially identified. 2,412 publications were excluded due to duplication, resulting 28,178 publications being analyzed. For LDA model, 6,250 publications were further excluded manually due to non-English language or incomplete abstract, resulting in 21,928 publications.

**Figure 2:**
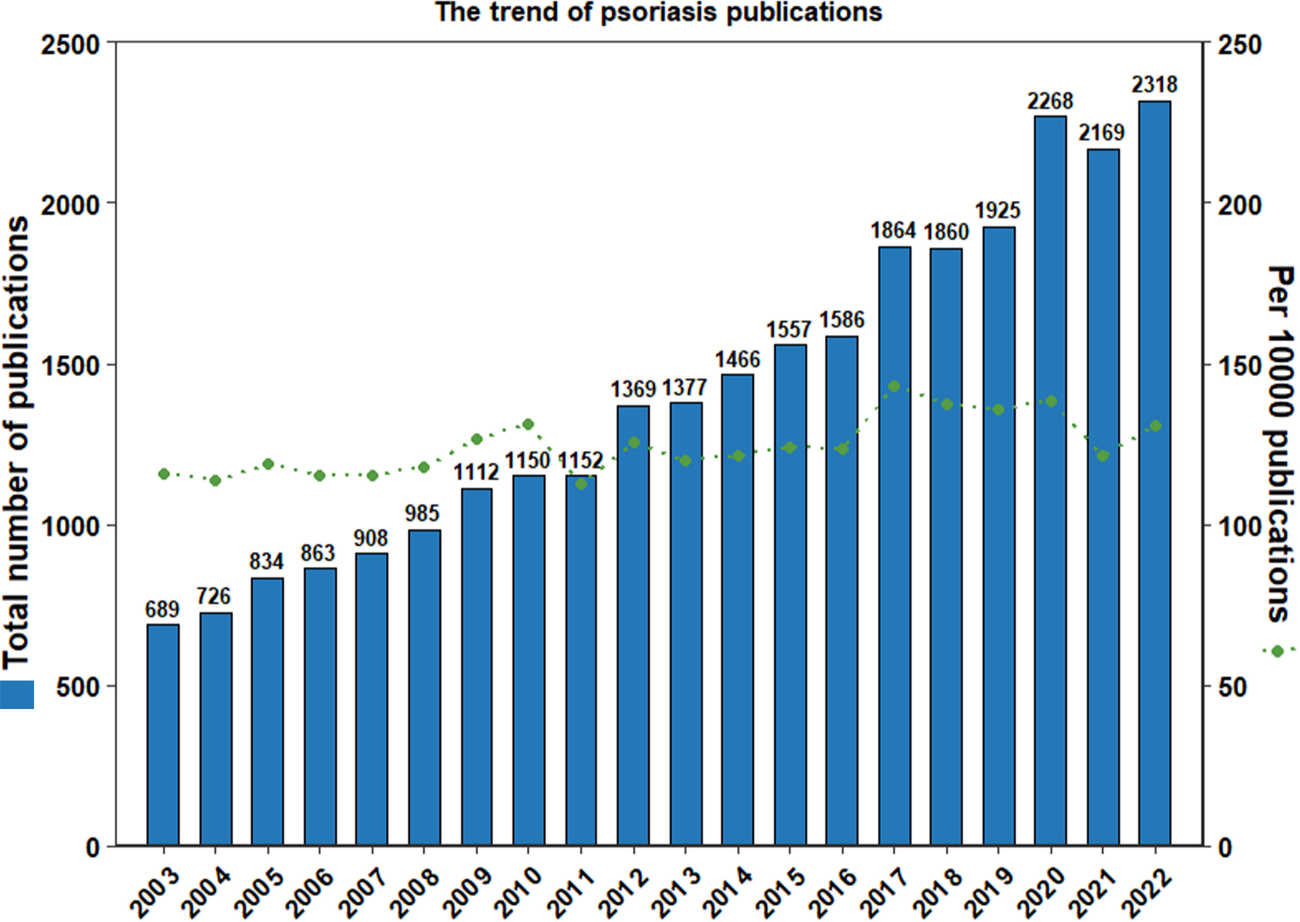
The number of publications on psoriasis has increased rapidly in the past 20 years. The numbers of publications on psoriasis each year was shown by blue columns, and the number of publications on psoriasis per 10,000 publications were shown by green spots. The number of publications increased from 689 in 2003 to 2,318 in 2022, with an average of 1408 publications published each year and an average growth rate by 6.25%.

From 2003 to 2022, 30,590 publications were identified under the MeSH term “Psoriasis”, as shown by a flowchart (**Figure 1**). After 2,412 publications being excluded for duplications, the remaining 28,178 publications were analyzed. For LDA model analysis, the number of the residual publications was further diminished by the exclusion of non-English publications or publications with incomplete abstracts, resulting in 21,928 remaining publications. 689 publications on psoriasis research were identified in 2003. The number of publications increased to 2,268 in 2020, 2,169 in 2021, and 2,318 in 2022. The details were shown in **Figure 2**. In the past 20 years, an average of 1408 publications were published each year, with the average growth rate by 6.25%.

### The United States, China, and Italy have the highest numbers of publications in psoriatic research

**Figure 3:**
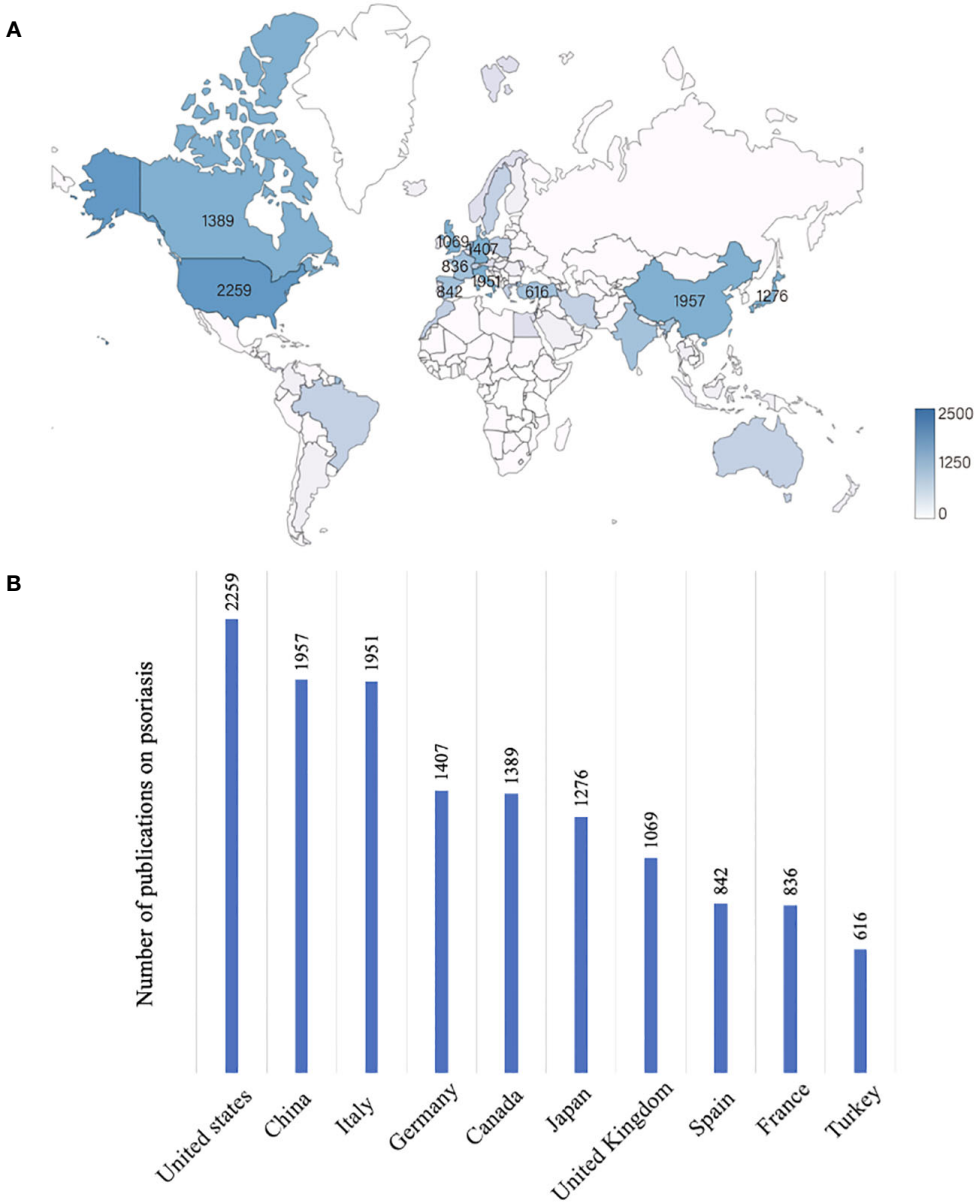
Global research on psoriasis differs significantly between countries and regions. **(A)** The global distribution of publications on psoriasis in the last 20 years. The darker the color is, the greater the volume of publications. **(B)** Top 10 countries with the highest publication numbers in psoriatic research.

The information of authors and affiliations were analyzed to describe the geographical distribution of the publications on psoriatic research in the last 20 years. We found that 176 countries or regions had published articles on psoriasis (**Figure 3A**). Of all publications, the United States, China, Italy, Germany, and Canada accounted for 8.0%, 6.9%, 6.9%, 5.0%, and 4.9%, respectively. The top 10 countries with the highest number of publications account for 48.2% of all publications (**Figure 3B**).

### MeSH term “Severity of illness index”, “Treatment outcome”, “Dermatologic agents” occurs most frequently

**Figure 4:**
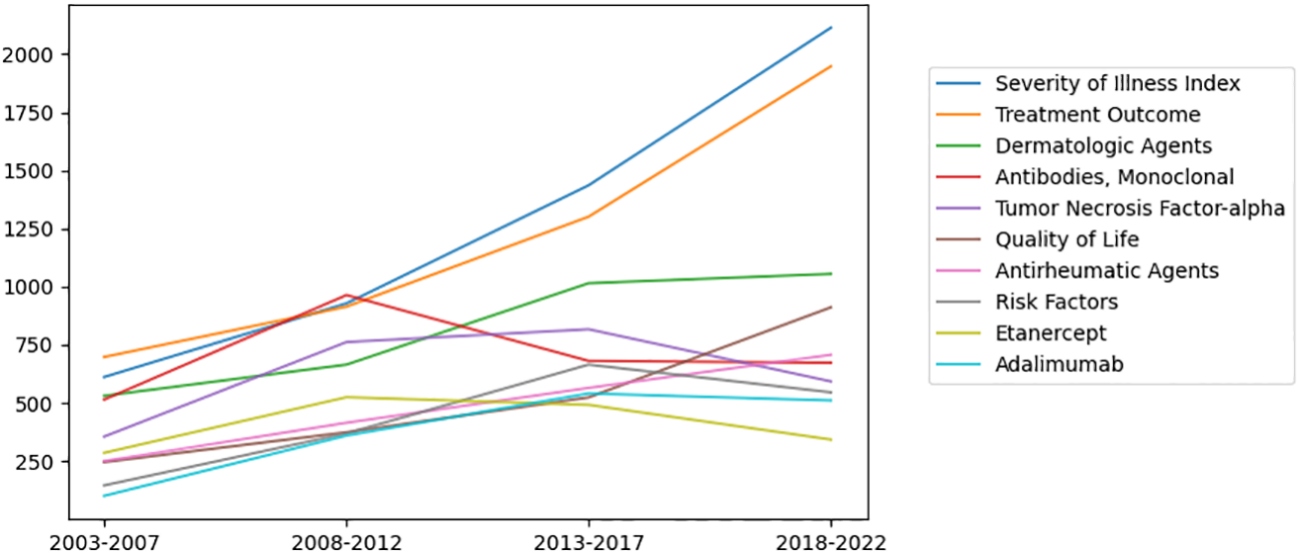
Ten of the most widely studied MeSH terms and their number of publications per year in the last 20 years. “Severity of illness index”, “Treatment outcome”, “Dermatologic agents”, “Antibodies, monoclonal” and “Tumor necrosis factor-alpha” were the top 5 MeSH terms with highest frequency.

To identify the research trends in a more detailed way, 9,183 MeSH terms related to psoriasis research with a total of 337,545 repetitions were analyzed from 2003 to 2022. The top ten MeSH terms with the highest number of publications changes every five years (**Table 1**). “Severity of illness index”, “Treatment outcome”, “Dermatologic agents”, “Antibodies, monoclonal” and “Tumor necrosis factor-alpha” were the top 5 MeSH terms with highest frequency (**Figure 4**), indicating that these topics have been studied most intensively in the last 20 years. In order to further explore the research trends of different countries and regions, we summarized the top 20 hot MeSH terms of articles published by the United States, China, and Italy (**Table 2**). Researchers from the United States and Italy concentrated more on treatment study and biological agents, while those from China focused more on basic research of psoriasis.

**Table 1:** The top ten widely studied MeSH terms in psoriasis research changes every five years over the past 20 years.

2003-2007			2008-2012		2013-2017		2018-2022	
MeSH term		Number of publications	MeSH term	Number of publications	MeSH term	Number of publications	MeSH term	Number of publications
**Top 1**	Treatment Outcome	578	Severity of Illness Index	811	Severity of Illness Index	1271	Severity of Illness Index	1737
**Top 2**	Severity of Illness Index	544	Treatment Outcome	768	Treatment Outcome	1115	Treatment Outcome	1578
**Top 3**	Dermatologic Agents	382	Antibodies, Monoclonal	684	Dermatologic Agents	785	Quality of Life	841
**Top 4**	Antibodies, Monoclonal	367	Tumor Necrosis Factor-alpha	588	Tumor Necrosis Factor-alpha	694	Antibodies, Monoclonal, Humanized	802
**Top 5**	Tumor Necrosis Factor-alpha	269	Dermatologic Agents	492	Case-Control Studies	651	Dermatologic Agents	778
**Top 6**	Immunoglobulin G	243	Receptors, Tumor Necrosis Factor	400	Risk Factors	575	Retrospective Studies	729
**Top 7**	Immunosuppressive Agents	233	Etanercept	394	Antibodies, Monoclonal, Humanized	529	Biological Products	683
**Top 8**	Receptors, Tumor Necrosis Factor	229	Case-Control Studies	363	Quality of Life	489	Interleukin-17	664
**Top 9**	Quality of Life	220	Quality of Life	336	Retrospective Studies	467	Inflammation	593
**Top 10**	Etanercept	217	Risk Factors	319	Antirheumatic Agents	466	Keratinocytes	556

MeSH, the Medical Subject Headings.

**Table 2:** The top 20 MeSH terms with most publications of United States, China, and Italy from 2003 to 2022.

Country	United States		China		Italy	
	MeSH term	Number of publications	MeSH term	Number of publications	MeSH term	Number of publications
**Top 1**	Treatment Outcome	431	Keratinocytes	337	Treatment Outcome	460
**Top 2**	Severity of Illness Index	406	Disease Models, Animal	260	Severity of Illness Index	414
**Top 3**	Tumor Necrosis Factor-alpha	309	Case-Control Studies	234	Antibodies, Monoclonal	333
**Top 4**	Antibodies, Monoclonal, Humanized	284	Cell Proliferation	224	Antibodies, Monoclonal, Humanized	313
**Top 5**	Antibodies, Monoclonal	248	Signal Transduction	213	Dermatologic Agents	298
**Top 6**	Antirheumatic Agents	208	Severity of Illness Index	210	Tumor Necrosis Factor-alpha	245
**Top 7**	Dermatologic Agents	205	Cytokines	206	Etanercept	204
**Top 8**	Etanercept	201	Inflammation	196	Immunoglobulin G	183
**Top 9**	Retrospective Studies	200	Genetic Predisposition to Disease	168	Receptors, Tumor Necrosis Factor	178
**Top 10**	Adalimumab	172	Interleukin-17	167	Double-Blind Method	169
**Top 11**	Quality of Life	142	Treatment Outcome	165	Quality of Life	168
**Top 12**	Biological Products	139	Tumor Necrosis Factor-alpha	126	Interleukin-17	159
**Top 13**	Immunoglobulin G	135	Polymorphism, Single Nucleotide	119	Immunosuppressive Agents	158
**Top 14**	Case-Control Studies	131	Gene Expression Regulation	110	Antirheumatic Agents	149
**Top 15**	Infliximab	131	RNA, Messenger	101	United States	134
**Top 16**	Receptors, Tumor Necrosis Factor	130	MicroRNAs	97	Inflammation	132
**Top 17**	Immunosuppressive Agents	127	Cells, Cultured	92	Adalimumab	126
**Top 18**	Arthritis, Rheumatoid	122	NF-kappa B	91	Risk Factors	116
**Top 19**	Risk Factors	121	Risk Factors	90	Retrospective Studies	115
**Top 20**	Aged, 80 and over	121	Interleukins	88	Biological Products	111

MeSH, the Medical Subject Headings.

### LDA identified three areas and 50 branched topics in psoriatic research

**Figure 5:**
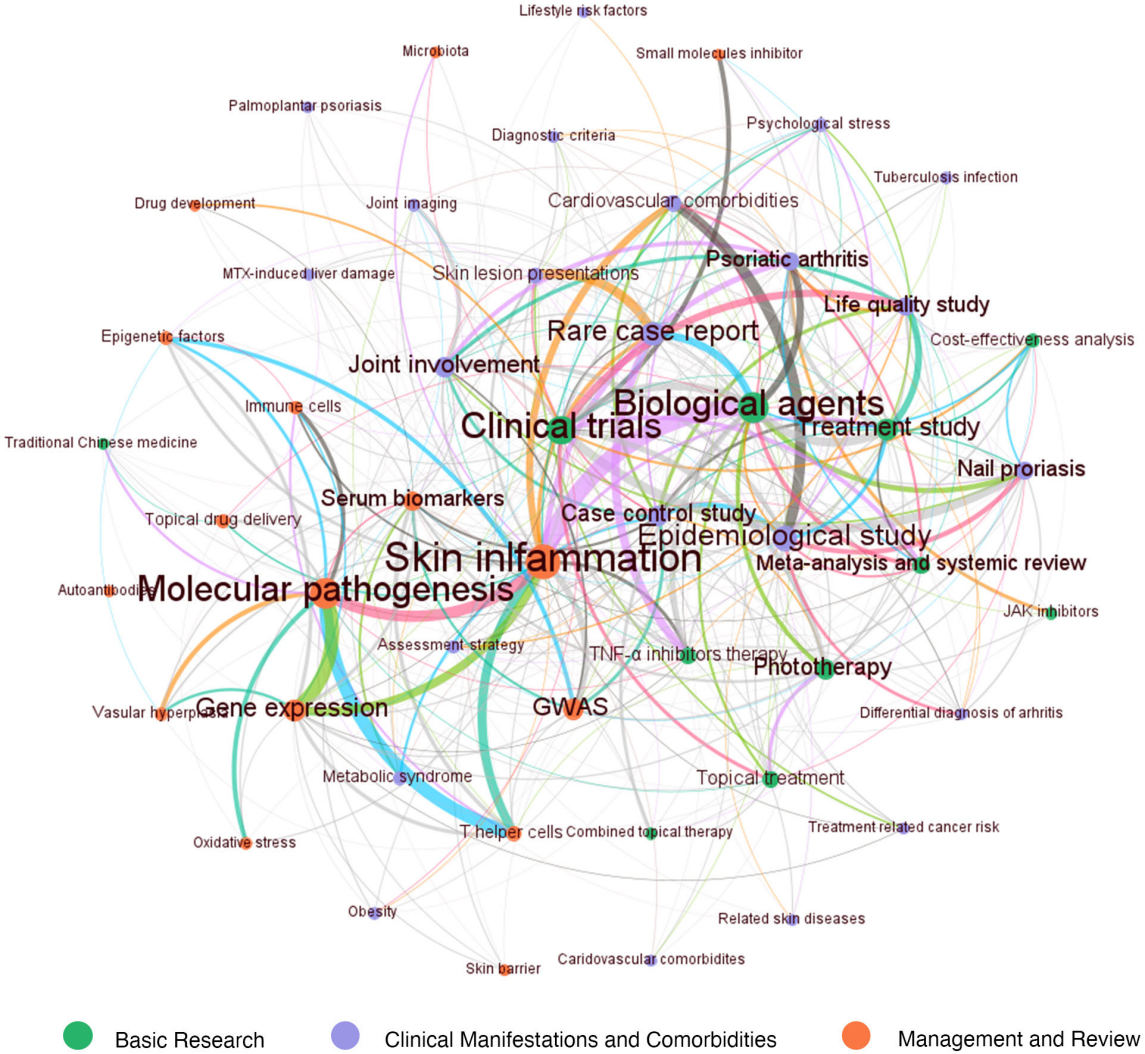
The three main areas and 50 topics in psoriasis research over the last 20 years identified by LDA algorithm. The 50 topics were classified into three broad categories: “Basic research (orange-marked)”, “Management and review (green-marked)”, and “Clinical manifestations and comorbidities (purple-marked)”. The number of publications on each topic was represented by circle size. The correlation between two topics was represented by a line between two circles, and the line thickness indicated the degree of overlap.

**Figure 6:**
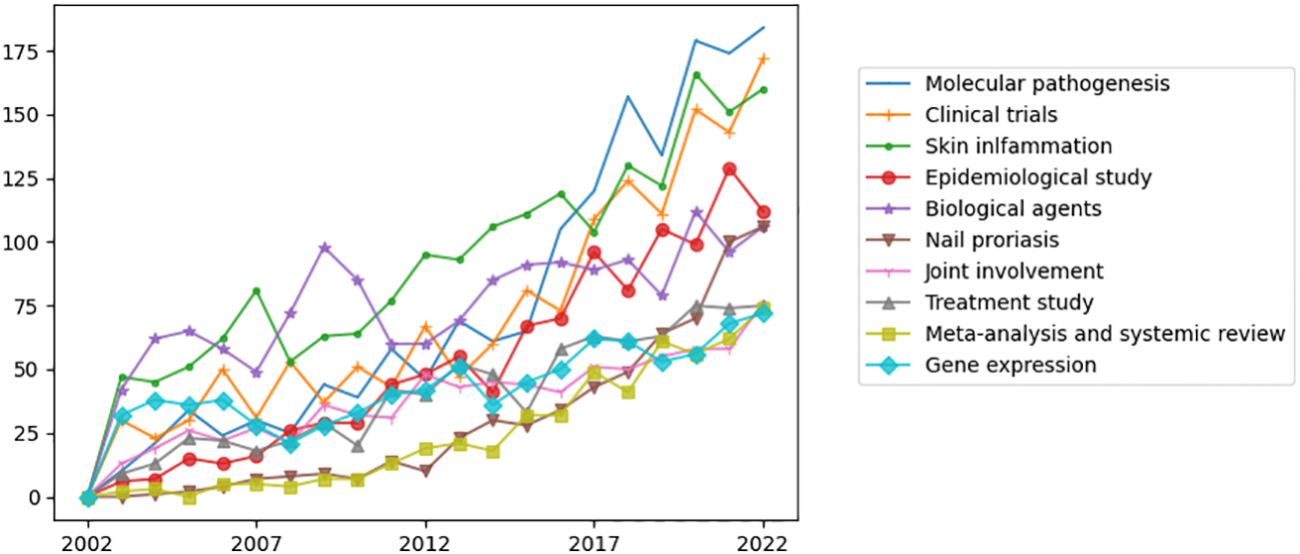
The top ten widely studied topics over the past 20 years identified by LDA algorithm. “Molecular pathogenesis”, “Clinical trials”, and “Skin inflammation” being the top three most studied topics among the 50 topics identified by LDA algorithm.

LDA algorithm was used to identify the main topics in psoriasis research in a more intuitive way compared to MeSH terms. The essential topics over the past 20 years and the relationships between them were demonstrated in a network built by LDA and Louvain algorithm. We manually classified the 50 topics into the following three areas: “Basic research”, “Management and review”, and “Clinical manifestations and comorbidities” (**Figure 5**). Each main topic was associated with several branches, which were indicated by circles with the same color. The circle size indicated the number of publications on this topic. The relationships between two topics were indicated by the thickness of lines. “Molecular pathogenesis”, “Clinical trials”, and “Skin inflammation” being the top three most studied topics among the 50 topics identified by LDA algorithm (**Figure 6**).

Firstly, the orange-marked area “Basic research” in **Figure 5** can be subdivided into the branches “Skin inflammation”, “Molecular pathogenesis”, “Gene expression”, “GWAS (genome-wide association studies)”, “Serum biomarkers”, “T helper cells”, “Oxidative stress”, “Vascular hyperplasia”, “Antibodies”, “Topical drug delivery”, “Immune cells”, “Epigenetic factors”, and “Drug development”. Within this topic, “Skin inflammation” was strongly associated with “Molecular pathogenesis”, “T helper cells” and “Gene expression”. “Skin inflammation” showed a strong connection with “Biologic agents” in green-marked cluster “Management and review” and “Psoriasis arthritis” and “Cardiovascular comorbidities” in purple-marked cluster “Clinical manifestations and comorbidities”. Thus, the “Skin inflammation” may play an important role in combining clinical manifestations and treatment study.

Secondly, another main area “Management and review” was demonstrated by green-labeled circles. This topic can be divided into “Biological agents”, “Clinical trials”, “Treatment study”, “phototherapy”, “Topical treatment”, “TNF-α inhibitors therapy”, “JAK (Janus kinase) inhibitors”, “Combined topical therapy”, “Cost-effectiveness analysis”. “Biologic agents” had strong connections with “Psoriasis arthritis”, “Nail psoriasis”, and “Rare case report”. Also, it was significantly associated with “Meta-analysis and systemic review”.

Thirdly, the purple-marked circles indicated the area “Clinical manifestations and comorbidities”. The main branches included “Epidemiological study”, “Rare case report”, “Case control study”, “Joint involvement”, “Psoriasis vulgaris”, “Nail psoriasis”, and “Life quality study”. Besides, this topic also covered some small branches, including “Metabolic syndrome”, “Skin lesion presentations”, “Palmoplantar psoriasis”, “Joint imaging”, “Differential diagnosis of arthritis”, “Diagnostic criteria”, “Obesity”, “Psychological diseases”, “Tuberculosis infection”, “Lifestyle risk factors”, “Related skin diseases”, “Treatment-related cancer risk”, and “MTX (methotrexate)-induced liver damage”. “Epidemiological study” was tightly connected with “Cardiovascular comorbidities”, “Case control studies”, “Nail psoriasis” and “Differential diagnosis of arthritis”.

### LDA heatmap results: “Molecular pathogenesis”, “Clinical trials” and “Skin inflammation” are current focuses and most increased topics

**Figure 7:**
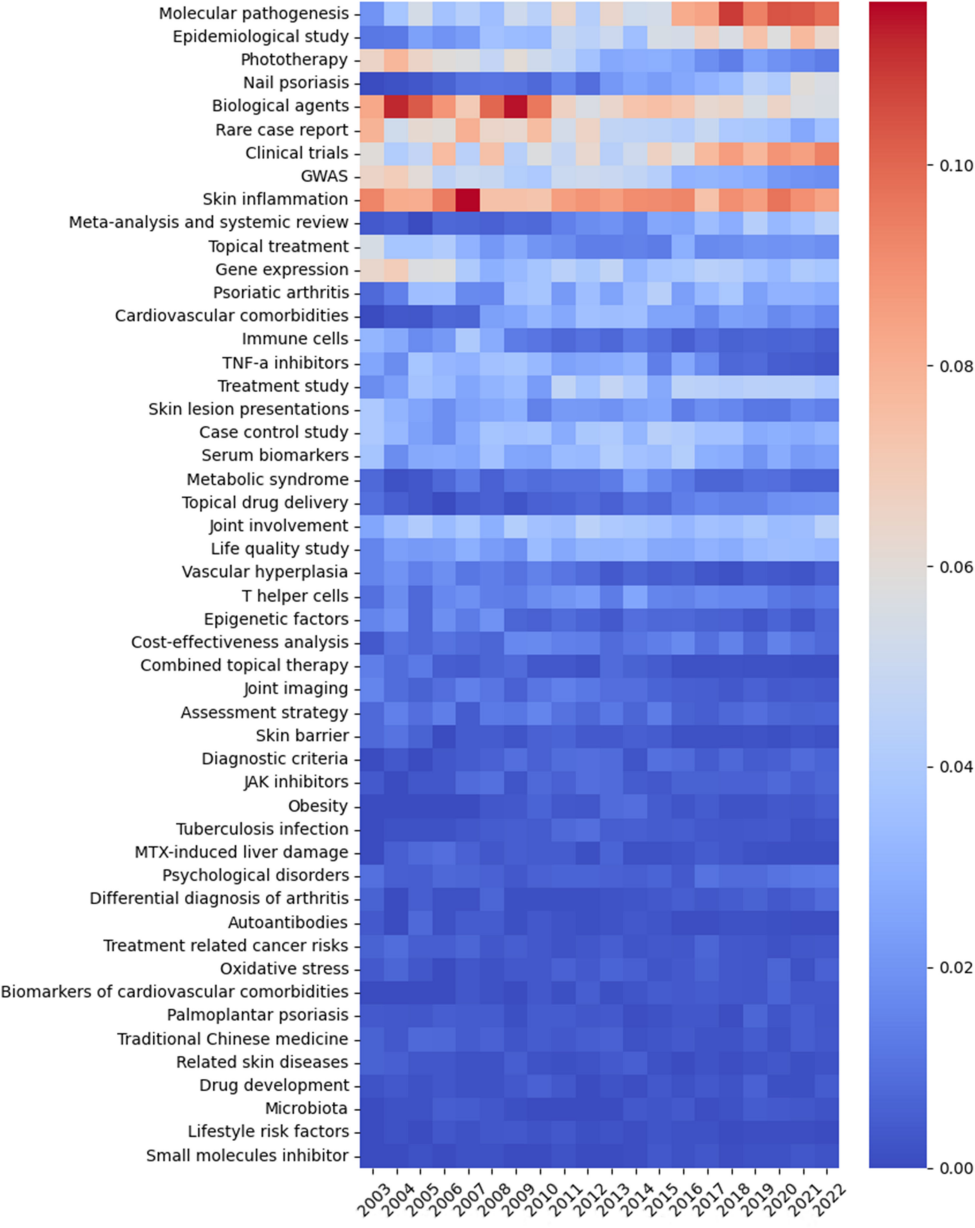
The heatmap presenting the normalized number of publications per year of 50 topics identified by LDA algorithm over the past 20 years. The abscissa represents the year, the ordinate represents the topics, and the color brightness represents the normalized number of publications.

To reflect the current trends of research topics in psoriasis, we visualized the LDA results by a heatmap that shows the normalized number of publications of 50 research topics per year (**Figure 7**). We identified “Molecular pathogenesis”, “Clinical trials”, and “Skin inflammation” to be the top three topics drawing the most concentration and the most increased three topics over the past 20 years. “Biological agents” has been a study focus for a long time, and the number of publications has been gradually increased in the last three years. “Nail psoriasis” and “Epidemiological study” have presented as new research hotspots in past three years. Topics on comorbidities of psoriasis, including “Cardiovascular comorbidities”, “Psoriatic arthritis”, “Obesity” and “Psychological disorders” have increased gradually. Instead, the attention to “TNF-α inhibitors”, “Combined topical therapy” and “MTX-induced liver damage” has decreased.

## Discussion

In this study, we analyzed 21,928 articles published from January 2003 to November 2022 in the field of psoriasis by employing machine learning and NLP. We demonstrated the hotspots and trends from a macroscopic perspective. To the best of our knowledge, this is the first study to take an overall picture of psoriatic research. In the past 20 years, the number of publications on psoriasis increased from 689 in 2003 to 2,318 in 2022. VOSviewer, Bibliographic Items Cooccurrence Matrix Builder (BICOMB), and CiteSpace can assist bibliometric analysis. However, the analysis conducted by these tools is based on keywords and is restricted by the limited data processing capabilities. Thus, we used LDA, an unsupervised machine learning method, to deal with the metadata consisting of 21,928 articles.

When considering the total number of publications over the past 20 years, we found “Severity of illness index”, “Treatment outcome”, and “Dermatologic agents” were the most concentrated MeSH terms, indicating the rapid therapeutic development in psoriasis. Consistently, LDA identified the top five areas were “Skin inflammation”, “Molecular pathogenesis”, “Biologic agents”, “Clinical trials” and “Epidemiological study”. Among them, “Skin inflammation” showed a strong connection with “Biologic agents” and a moderate connection with “Clinical trials”, indicating the effective translation between basic research and clinical application in psoriasis. Over the past decades, we have made great progress in elucidating the occurrence and development of psoriasis. Numerous effective and well-tolerated medications, particularly pathogenesis-oriented biologic agents have emerged. It has been widely recognized that the interaction between components of the innate and adaptive immune system is the central part in the development of psoriasis, which is mediated by various cytokines, chemokines and other mediators (1, 17). Our study found that Tumor Necrosis Factor-alpha has consistently been in the top 10 MeSH terms from 2003 to 2017. However, in the past 5 years, Interleukin-17 seemed to replace Tumor Necrosis Factor-alpha and entered the top 10 Mesh Terms (**Table 1**). This change may be resulted from the evolution of recognizing psoriasis as a T helper 1 (Th1)/T helper 17 (Th17)-mediated disease. Biologics targeted at tumor necrosis factor alpha (TNF-α) have been available for the treatment of psoriasis for more than 18 years (18, 19), with Etanercept being the first agent approved by the United States (US) Food and Drug Administration (FDA) in 2004. The other group of agents which targeted at IL-23/Th-17 axis has been available for more than 13 years, with the target changing from IL-12/IL-23 p40 to IL-17 (2, 19–22). Early in 2004, IL-17 messenger RNA (mRNA) has been reported to be observed in psoriatic lesions (23, 24). In 2008, Th17 was identified as a distinct cell population in the dermis of psoriatic lesions (25). The association between accumulation of IL-17 mRNA and disease activity indicated IL-17 could be proximal regulators of skin inflammation (25). This hypothesis was further supported by the clinical study in 2010 (26). Since then, accumulating evidence has shown the excellent results of biologic agents that specifically target IL-17 and IL-17 receptor (2). Secukinumab was finally approved in January 2015 both by US FDA and European Medicines Agency (EMA) (2). These therapeutic developments enable many patients with moderate to severe psoriasis being treated effectively, even to the point of complete remission (22). However, the situation is not the same for mild patients, for whom effective systemic medications are not routinely approved (27). Besides, there is also a need for effective and safe treatments for non-plaque psoriasis, including pustular psoriasis and erythrodermic psoriasis which have been recognized as a distinct entity (28–30).

There are mainly two directions for therapeutic development in psoriasis research (27). The first approach is to target different and additional mediators, which is based on the more intensive elucidation of pathogenesis through basic research. Based on the observation that the higher levels of IL-17F was seen in skin lesions than unaffected parts of the skin (31), bimekizumab, which blocks IL-17A as well as IL-17F, has been approved in 2021 by European Medicines Agency (EMA) and presents with convincing efficacy (32). Besides, as the IL-36 axis has been found to play a critical role in the autoinflammatory responses in pustular psoriasis (33), spesolimab and imsidolimab, which inhibit IL-36 pathway, are currently developed (34). Spesolimab has been approved by FDA for generalized pustular psoriasis in 2022 (35). Imsidolimab has also shown promising results in clinical trials (36). The second way is to target small molecules in the central signaling pathways. Apremilast, a phosphodiesterase 4 inhibitor, is the first small-molecule inhibitor approved for psoriasis treatment (37). The family of JAK is another focus, which mediates intracellular signaling after activation of type I and type II cytokine receptors (38). The JAK1/3 inhibitor tofacitinib and the selective JAK1 blocker upadacitinib have been approved in psoriatic arthritis (PsA) (39, 40). Tyrosine kinase 2 inhibitor deucravacitinib was approved for oral therapy of moderate-to-severe psoriasis in the United States in September 2022 (41). Besides, Piclidenoson, a Gi protein-associated A_3_ adenosine receptor (A_3_AR) agonist, which in turn inhibit NF-*κ*B activity, is in early phase of development (42, 43).

Of note, the number of publications about the MeSH term Quality of Life has significantly increased and came third just behind Severity of Illness Index and Treatment outcome in the period of 2018-2022. Most studies evaluating treatment outcomes focus on objective evaluation of skin lesions as the primary endpoint of efficacy (44). However, it has been recognized that psoriasis also brings patients great burden on life quality (17, 44). The damage to life quality of patients from psoriasis was similar to or even worse than that from other chronic diseases, including diabetes mellitus, ischemic heart disease, asthma or bronchitis (45). Besides, the correlation between the most widely used Psoriasis Area Severity Index (PASI) and Dermatology Life Quality Index (DLQI) has been reported to be relatively low in some studies (46–48). Therefore, measurements of life quality have become a necessary adjunct to traditional clinical assessments (44), resulting in the increased number of publications about Quality of Life in psoriatic research.

Next, when exploring the trends and current hotspots of psoriatic research, we identified “Nail psoriasis” and “Epidemiological study” have presented as new research hotspots following “Molecular pathogenesis”, “Clinical trials”, and “Skin inflammation”. Nail psoriasis, which impairs the manual function, is seen in 50–79% of patients with skin psoriasis and up to 80% of patients with PsA (49). Recently, continuous effort has been made in assessment methods, diagnosis, differential diagnosis, and treatment of nail psoriasis. However, to date, there is no standard treatment algorithm of nail psoriasis (50). Topical therapy is the first-line option in mild nail psoriasis (50, 51). Biologic agents are recommended to patients with more than three nails involved, with severely damaged quality of life, or suffered from moderate-to-severe skin psoriasis or PsA (50, 51). Although biologic agents have shown more promising efficacy compared to other therapies, more data are needed to thoroughly evaluate the efficacy and safety (50–52). Epidemiological studies concentrate on the disease in populations, rather than in individuals, with disease causation being the explicit study aim (53). Accumulating epidemiological studies have demonstrated that the prevalence of psoriasis varies with the country (3), indicating that ethnicity, genetic background, and environmental factors affect the onset of psoriasis. Beyond genetic susceptibility, the risk factors for psoriasis can be divided into extrinsic and intrinsic risk factors (54). The intrinsic group include MetS, obesity, diabetes mellitus, dyslipidemia, hypertension and mental disorders. The other extrinsic group consists of mechanical stress, air pollution, drugs, vaccination (COVID-19 vaccination (55), influenza vaccination (56), tetanus-diphtheria vaccination (57)), infection, smoking and alcohol (54).

It has become a consensus that psoriasis is not just a skin disease. Instead, psoriasis has been recognized as a systemic inflammatory disease (1, 58, 59), associated with an increased risk of MetS (59), CVD (60, 61), psychological diseases (62), inflammatory bowel disease (63), and cancer (64). Our study demonstrated that topics on comorbidities of psoriasis, including “Cardiovascular comorbidities”, “Psoriatic arthritis”, “Obesity” and “Psychological disorders” have gradually drawn increased attention in the past 20 years. Due to the worse prognosis, heavier financial burden and poorer life quality brought by comorbidities, tremendous attention has been drawn from researchers and clinicians (65, 66). Among these comorbidities, CVD have aroused great interest of clinicians due to the high mortality (61). A large number of observational studies and meta-analysis showed that patients with psoriasis have a higher risk of CVD (67–69). Myocardial infarction and stroke have been more frequently observed in patients with psoriasis than in the general population, particularly in people with severe psoriasis (70–72). A meta-analysis and mendelian randomization analysis in 2022 identified causality between psoriasis and CVD, and suggested that management of psoriasis might be beneficial to cardiovascular outcomes (73). MetS is one of the most common comorbidities of psoriasis (65), representing a cluster of metabolic abnormalities, including obesity, hypertension, diabetes mellitus, hyperlipidemia, and obesity-associated non-alcoholic fatty liver disease (74). MetS not only directly increases the risk of CVD and premature mortality in patients (75), but also reduces the response to biologic agents of psoriatic patients (76). Psychological diseases, mainly anxiety and depression, are found to be triggers both for the occurrence, progression, and relapse of psoriasis. Depending on different screening tools, 9.9%-62% psoriatic patients suffered from depression (77–79), and 7-48% patients suffered from anxiety (78, 80). Several mechanisms have been hypothesized to explain the association between psoriasis and comorbidities, including sharing same risk factors, overlapped genetic background, and common inflammatory pathways (Th1 and Th17 pathways). However, the accurate pathogenesis remains unknown.

Based on the hypothesis that the association was partly caused by overlapped inflammatory pathways, it is interesting to determine whether anti-psoriatic treatment would have simultaneous protective effect on comorbidities. The impact from biologic agents on CVD risk was the mostly studied topic, and some studies demonstrated positive improvement to CVD risk by biologic agents (81–83). Epidemiological studies suggested TNF-α inhibitors were associated with a reduced risk of cardiovascular events compared to topical therapy/phototherapy (84) and methotrexate (85). IL-12/23 inhibitors were found to improve the left ventricular strain, arterial stiffness and coronary microcirculation estimated by LAD doppler echocardiography (86). However, other studies found neutral or negative impacts. TNF-α inhibitors was associated with heart failure risk, and thus is not recommended in psoriatic patients whom have a known history of heart failure (87). IL-17 inhibitors was observed to have no effect on aortic vascular inflammation assessed by Fluorodeoxyglucose positron emission tomography computed tomography (FDG PET/CT) and cardiometabolic disease biomarkers after 52 weeks of treatment (88). An IL-12/23 inhibitor (Briakinumab) was reported to have an increase in major adverse cardiovascular events after phase III trials (89, 90). Further prospective studies are needed, and it may be more efficient to manage the conventional CVD risk factors (hypertension, dyslipidemia, and type 2 diabetes) in patients with psoriasis (61).

There were some limitations of the present study. Firstly, as analysis was based on existing publications, the ability to predict future directions of psoriasis research was limited. Secondly, although we analyzed the text information from titles and abstracts with the assistance of LDA methods, there still may be some bias due to not including full texts of publications into analysis. Thirdly, for the reason that the reliability of evidence provided by case reports and other articles is of difference, there could be some bias for including case reports in the analysis despite they only accounted for 8.2%.

## Conclusion

The number of publications in psoriatic research has increased rapidly during the last 20 years. “Severity of illness index”, “Treatment outcome”, and “Dermatologic agents” were the most concentrated MeSH terms over the past 20 years. Skin inflammation, molecular pathogenesis, biological agents, clinical trials, epidemiological study, nail psoriasis, and psoriasis comorbidities, are current research hotspots identified by LDA. The strong connection between skin inflammation and biologic agents identified by Louvain algorithm indicated an effective translation between basic research and clinical application in psoriasis. Bibliometric analysis assisted by machine learning method may be a potential powerful tool for scientists to quickly grasp the current status and hotspots from a macroscopic view on psoriatic research.

## Data availability statement

The original contributions presented in the study are included in the article/supplementary material. Further inquiries can be directed to the corresponding authors.

## Author contributions

CY: Conceptualization, Data curation, Formal Analysis, Methodology, Writing – original draft, Writing – review & editing. YH: Conceptualization, Data curation, Formal Analysis, Methodology, Software, Validation, Visualization, Writing – original draft. WY: Conceptualization, Supervision, Writing – review & editing. XJ: Conceptualization, Funding acquisition, Supervision, Writing – review & editing.
